# The use of computerized echocardiographic simulation improves the learning curve for transesophageal hemodynamic assessment in critically ill patients

**DOI:** 10.1186/s13613-016-0132-x

**Published:** 2016-04-07

**Authors:** Gwénaël Prat, Cyril Charron, Xavier Repesse, Pierre Coriat, Pierre Bailly, Erwan L’her, Antoine Vieillard-Baron

**Affiliations:** Medical Intensive Care Unit, University Hospital of Brest, Cavale Blanche, 29609 Brest Cedex, France; Centre de Simulation en Santé, Université de Bretagne Occidentale, Brest, France; Intensive Care Unit, Section Thorax-Vascular Disease-Abdomen-Metabolism, Assistance Publique-Hôpitaux de Paris, University Hospital Ambroise Pare, 9, Avenue Charles-de-Gaulle, 92100 Boulogne-Billancourt, France; Hospital Pitié-Salpêtrière, Department of Anesthesiology and Critical Care, Assistance Publique-Hôpitaux de Paris, University Pierre et Marie-Curie-Paris 6, Paris, France; LATIM INSERM UMR 1101, Université de Bretagne Occidentale, Brest, France; FHU TechSan, Université de Bretagne Occidentale/Université de Rennes, Rennes, France; INSERM U-1018, CESP, Team 5 (EpReC, Renal and Cardiovascular Epidemiology), UVSQ, 94807 Villejuif, France

**Keywords:** Education, Transesophageal echocardiography, Critical care, Echocardiography, Mannequin echocardiographic simulator

## Abstract

**Background:**

Our aim was to evaluate the impact of a computerized echocardiographic simulator on the learning curve for transesophageal echocardiography (TEE) hemodynamic assessment of ventilated patients in the ICU.

**Methods:**

We performed a prospective study in two university hospital medical ICUs. Using our previously validated skill assessment scoring system (/40 points), we compared learning curves obtained with (interventional group, *n* = 25 trainees) and without (control group, *n* = 31 trainees) use of a simulator in the training. Three evaluations were performed after 1 (M1), 3 (M3) and 6 months (M6) while performing two TEE examinations graded by an expert. Competency was defined as a score >35/40.

**Results:**

Competency was achieved after an average of 32.5 ± 10 supervised studies in the control group compared with only 13.6 ± 8.5 in the interventional group (*p* < 0.0001). At M6, a significant between-group difference in number of supervised TEE was observed (17 [14–28] in the control group vs. 30.5 [21.5–39.5] in the interventional group, *p* = 0.001). The score was significantly higher in the interventional group at M1 (32.5 [29.25–35.5] vs. 24.75 [20–30.25]; *p* = 0.0001), M3 (37 [33.5–38.5] vs. 32 [30.37–34.5]; *p* = 0.0004), but not at M6 (37.5 [33–39] vs. 36 [33.5–37.5] *p* = 0.24).

**Conclusion:**

Inclusion of echocardiographic simulator sessions in a standardized curriculum may improve the learning curve for hemodynamic evaluation of ventilated ICU patients.

**Electronic supplementary material:**

The online version of this article (doi:10.1186/s13613-016-0132-x) contains supplementary material, which is available to authorized users.

## Background

Since the early 1960s and the first resuscitation manikin, technological improvements have allowed the development of virtual-reality training simulators [1, 2]. In the surgical field, the impact of 3D haptic laparoscopy simulators has been extensively explored, and the transferability of bedside skills after simulation training [3–5] has been validated, leading to a European consensus on a competency-based virtual-reality training program [6].

Critical care echocardiography (CCE) has gained acceptance and in its basic form based on transthoracic echocardiography (TTE) is now recommended for inclusion in the curriculum of all intensivists [7, 8]. Advanced CCE is also recognized as a way to monitor hemodynamics fully in the ICU [9], but requires acquisition of technical skills in transesophageal echocardiography (TEE). Traditionally, after a standard curriculum using didactic courses, trainees acquire technical skills at the bedside, under the supervision of experienced physicians. International consensus statements establish how physicians can acquire the competency needed for ICU practice [8, 9]. Types of echocardiographic views, measurements and the overall number of bedside TEE and TTE examinations needed to achieve competency are defined, but very little is mentioned about virtual-reality simulation [10]. To our knowledge, there is only one study reporting its inclusion in a cardiovascular curriculum, in terms of skills acquisition and proficiency [11].

In two previous studies we validated a skills assessment scoring system and used it to demonstrate that at least 31 supervised bedside TEE examinations, performed during a 6-month period, were required to achieve competency in hemodynamic evaluation of ventilated ICU patients [12, 13]. The third part of this educational process is represented herein and is designed to evaluate the impact of a TEE virtual-reality simulator, integrated into the previously described curriculum.

## Methods

We conducted a prospective, multicenter study in two French university hospital medical ICUs between May 2012 and November 2014. The results for trainees trained without a simulator (control group) between November 2006 and June 2010 [13] were compared with those recorded during the second period between May 2012 and November 2014 with the simulator (interventional group). In the participating centers, TEE has been used for years as a first-line tool for hemodynamic assessment in critically ill patients. As a result, more than 350–450 TEE examinations are performed yearly in each center in mechanically ventilated patients presenting with shock or acute respiratory failure. Our study was therefore considered as part of routine practice by our local ethics committee, and no informed consent was required from the patients or their next of kin.

All volunteer residents rotating in our ICUs each 6 months, without previous experience in TEE, were consecutively included and constituted the evaluated population. Their previous experience in echocardiography was graded as level 0 (no experience at all) or level 1 (previous experience in TTE without TEE experience). Each trainee performed and interpreted TEE examinations online, under the supervision of an expert, and the number of supervised TEE examinations was prospectively recorded during a 6-month period, as previously done [12]. A 2-h didactic course on echocardiographic basic ultrasounds was performed by an expert for the two groups (control group and interventional group) at the beginning of each 6-month period. Trainees in the interventional group also had two 3-h individual sessions of practical hands-on training using an echocardiographic simulator (Vimedix CAE Healthcare Inc, Montréal, Canada or Heartworks Intensive Medical Ltd, London, UK) during the first 3 months. Simulator learning was focused on the acquisition of the main esophageal views used for hemodynamic evaluation at the bedside: the mid-esophagus long-axis view 0°–120°, the transgastric short-axis view 0°–110° and the upper-esophagus (great vessels) view 0°–90° (Additional file 1: Figure S1).

After 1 (M1), 3 (M3) and 6 months (M6), trainees were evaluated by a supervisor using our previously validated scoring system (Table 1) [12, 13]. Briefly, the maximum score is 40 points with four fields of skills to grade: practical skill (/14 points) reflecting the ability of the trainee to obtain standard TEE views, evaluation skill (/10 points) assessing the semiquantitative evaluation of right ventricular size, respiratory variations in the diameter of the superior vena cava, mitral and aortic regurgitation, and pericardial effusion, technical skill (/8 points) reflecting the ability of the trainee accurately to measure simple hemodynamic parameters (e.g., left ventricular ejection fraction, velocity–time integral of left ventricular outflow tract Doppler velocities), and finally the interpretation skill (/8 points), which referred to the trainee’s ability to summarize the information obtained by the TEE examination and to suggest adequate therapeutic changes accordingly.

**Table 1:** Four-part skills assessment scoring system

				Score
Practical skill				
Introduction of the probe	No	Problematic	Yes	/2
TE long-axis view at 0°	Not recorded	Not optimal	Optimal	/2
TE long-axis view at 120°	Not recorded	Not optimal	Optimal	/2
TG short-axis view at 0°	Not recorded	Not optimal	Optimal	/2
TG short-axis view at 120°	Not recorded	Not optimal	Optimal	/2
TE view of the base of the heart at 0°	Not recorded	Not optimal	Optimal	/2
TE view of the base of the heart at 90°	Not recorded	Not optimal	Optimal	/2
Total				/14
Evaluation skill				
Mitral regurgitation	None	Moderate	Marked to massive	/2
Aortic regurgitation	None	Moderate	Marked to massive	/2
Dilatation of right ventricle	None	Moderate	Marked	/2
Pericardial effusion	None	Non compressive	Compressive	/2
Variations in diameter of superior vena cava	None	Minimal	Large	/2
Total				/10
	Intensivist		Expert	
Technical skill				
E/A ratio				/2
LV FAC (%)				/2
Aortic VTI (cm)				/2
Pulmonary VTI (cm)				/2
Total				/8
Interpretation skill				
LV contractility	Normal	Moderately decreased	Greatly decreased	/2
Hypovolemia	No		Yes	/2
RV failure	No		Yes	/2
Treatment proposed	Wrong or incomplete		Right	/2
Total				/8
Final score/40				

*TE* transesophageal, *TG* transgastric, *FAC* fractional area change, *LV* left ventricle, *RV* right ventricle, *VTI* velocity–time integral

The supervisor was an expert in advanced CCE and a full-time intensivist with more than 10 years of ICU experience and >200 TEE/year examinations performed on mechanically ventilated patients. All evaluations were performed by the same expert in each center, and trainees did not receive any feedback or assistance while performing TEE until they wrote the echocardiographic report.

### Statistical analysis

Statistical analysis was performed using MedCalc (9030 Mariakerke, Belgium). Continuous variables were expressed as median [25–75 percentiles]. Between-group comparisons were performed using nonparametric tests. ANOVA was used to compare changes in score and to build the learning curve. We compared learning curves between the previous period with no simulator use in the training process, in which we included 31 residents [13], i.e., the control group, and the current period using the simulator, i.e., the interventional group. In the control group, we excluded from the analysis 10 trainees from our previous published study performed in 41 trainees [13] since in one center of this previous study no TEE simulator was available for the second period. We were then able to compare the performance of trainees in the two groups with the same process of evaluation, the same experts, as well as the same scoring system. In the two groups, competency was arbitrarily defined by a skills assessment score >35/40, as previously done [13].

## Results

Fifty-six trainees were included during the two periods, 31 in the control group and 25 in the interventional group. Trainee characteristics were similar in the two groups. All were residents in the interventional group, while 30 were residents and 1 was senior intensivist in the control group (*p* = 0.58). No difference was observed in trainees’ specialty of origin (*p* = 0.39). Most were anesthesiologists (62 %) and cardiologists (19 %) in the interventional group. Eight trainees (32 %) had echocardiography level 1 experience in the interventional group and 15 (48 %) in the control group (*p* = 0.38).

**Fig. 1:**
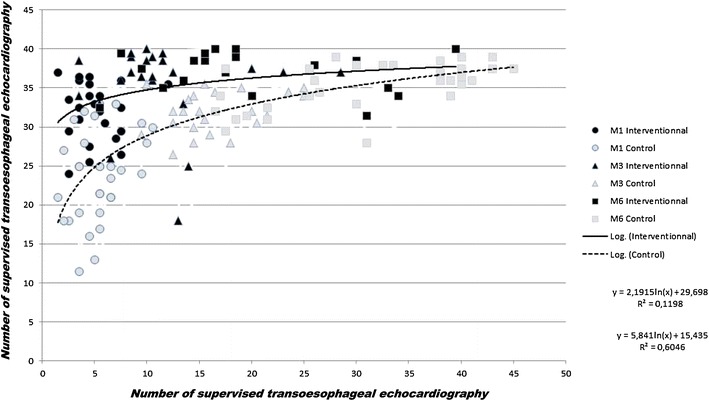
Correlation (Spearman’s coefficient of rank correlation) between the number of supervised TEE examinations performed by the trainees and the global skills assessment score obtained at M1, M3 and M6 in the control group (*dashed line*) and the interventional group (*full line*)

**Fig. 2:**
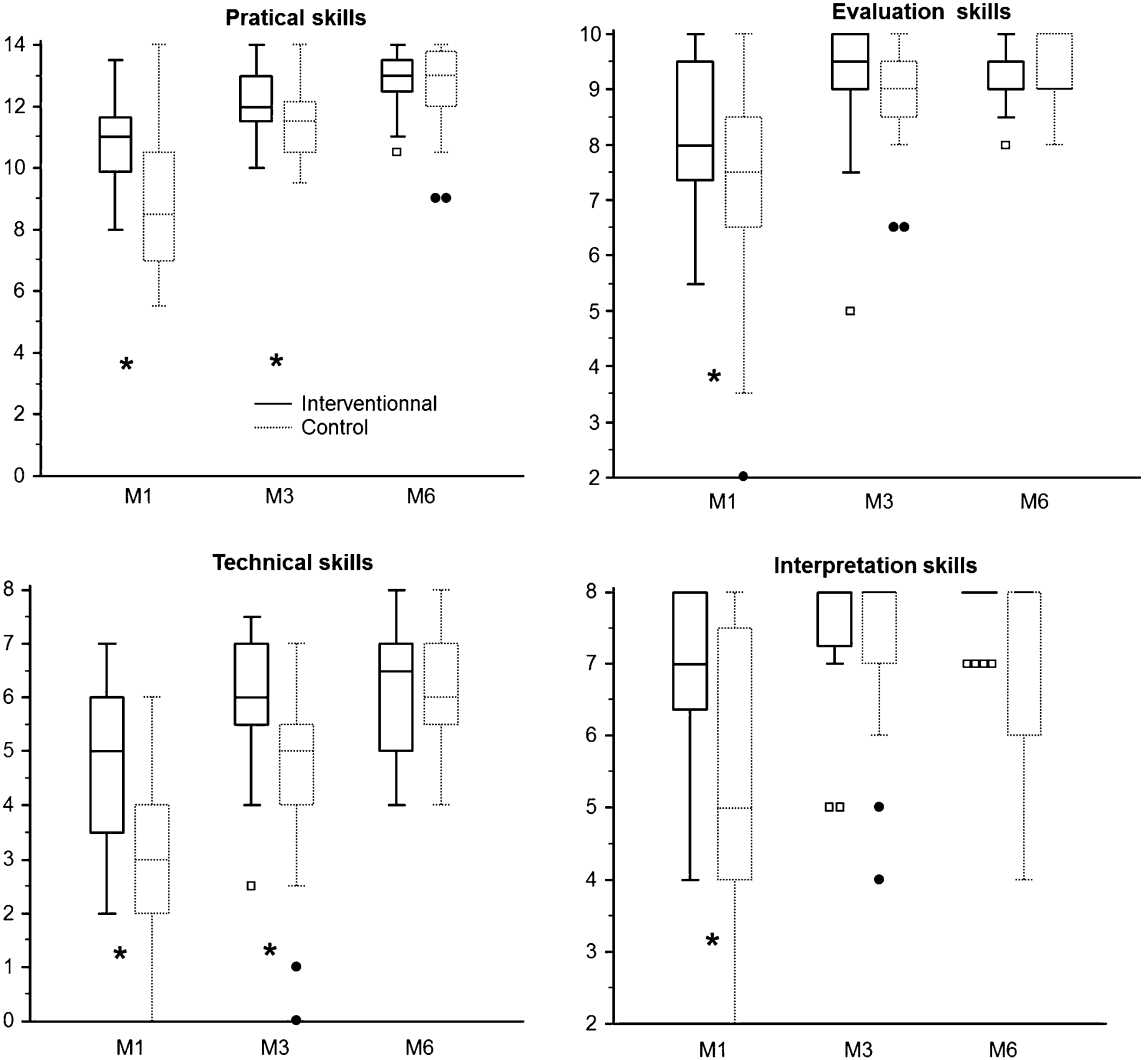
*Box* and *whisker plot* representation of the score (*y* axis) obtained in each part of the scoring system at 1 (M1), 3 (M3) and 6 months (M6) in the interventional and control groups. Median = *horizontal line inside the box*; upper and lower quartiles = *whisker plot*; *boxes* and *circles* represent values lower than the lower quartiles. *p < 0.05

The mean score improved faster over the 6-month period in the interventional group (*p* = 0.046, Fig. 1), with a significantly higher score at M1 and M3 although not at M6 (Table 2). Despite a significantly lower number of supervised TEE examinations at M6 (17 [14–28] versus 30.5 [21.5–39.5], *p* = 0.0004, Table 2), trainees in the interventional group reached the same average score as in the control group (37.5 [33–39] versus 36 [33.5–37.5], respectively, *p* = 0.24, Table 2). Competency was obtained after an average of 32.5 ± 10 supervised studies in the control group compared with 13.6 ± 8.5 in the interventional group (*p* < 0.0001). Comparison between each part of the scoring system is depicted in Table 3 and in Fig. 2. Practical and technical skills were significantly increased in the interventional group at M1 and M3, although not at M6, whereas no difference was observed for the evaluation and interpretation skills, except at M1.

**Table 2:** Skills assessment score

	M1	M3	M6	ANOVA
Mean score, interventional group	32.5 [29.25–35.5]	37 [33.5–38.5]	37.5 [33–39]	*p* = 0.048
Mean score, control group	24.75 [20–30.25]	32 [30.37–34.5]	36 [33.5–37.5]	
*p*	<0.0001	0.0004	0.24	
Number of supervised TEE examinations, interventional group	4.5 [3.5–6.25]	10.5 [8.5–13.25]	17 [14–28]	*p* = 0.001
Number of supervised TEE examinations, control group	5.5 [3.75–6.5]	15.5 [14–20]	30.5 [21.5–39.5]	
*p*	0.31	0.0003	0.0004	

Skills assessment score with the number of supervised TEE examinations at 1 (M1), 3 (M3) and 6 months (M6) in the interventional (with simulator) and control (without simulator) groups. ANOVA column corresponds to analysis of changes from M1 to M6, whereas p line relates to comparison between both groups at each evaluation. Data are expressed as median [25–75 percentiles]

*TEE* transesophageal echocardiography

**Table 3:** Evolution of the different parts of the scoring system after 1 (M1), 3 (M3) and 6 months (M6)

	M1		M3		M6	
	Interventional	Control	Interventional	Control	Interventional	Control
Practical skills (/14 pts)	11* [10–11.5]	8.5 [7–10.5]	12* [11.5–13]*	11.5 [10.5–12]	13 [12.5–13.5]	13 [12–14]
Evaluation skills (/10 pts)	8* [7.5 – 9.5]	7.5 [6.5–8.5]	9.5 [9, 10]	9 [8.5–9.5]	9.5 [9–9.5]	9 [9, 10]
Technical skills (/8 pts)	5* [3.5 – 6]	3 [2–4]	6* [5.5–7]	5 [4–5.5]	6.5 [5–7]	6 [5.5–7]
Interpretation skills (/8 pts)	7* [5, 6, 6–8]	5 [4–7.5]	8 [7, 8]	8 [7, 8]	8 [8]	8 [6–8]

Interventional group regards the group with a simulator and the control group the period without a simulator. Data are expressed as median [25–75 percentiles]

* *p* < 0.05 versus control group

At M1, M3 and M6 there was no between-group difference in the procedural time duration (interventional vs. control: 20 [17.5–24.5] vs. 19.5 min [17–25], 15 [12.5–18] vs. 14.75 min [13–18] and 12.75 [11.5–15.5] vs. 12 min [10–16], respectively).

## Discussion

Our study demonstrates that the addition of virtual-reality simulator sessions to a standardized 6-month curriculum improves the learning curve for ICU TEE hemodynamic assessment. The use of the simulator especially reduced the number of TEE examinations required to achieve competency by improving acquisition of practical and technical skills.

Virtual-reality simulators have proven to be an efficient addition to curricula in various healthcare specialties, including laparoscopy [14, 15], upper- and lower-gut endoscopy [16, 17] and endoscopic sinus surgery [18]. Most recent virtual-reality echocardiographic simulators are quite realistic, according to hands-on training or questionnaire evaluations [19, 20]. In a questionnaire study, a vast majority of participants answered that simulators are realistic, easy-to-use and helpful for image acquisition and interpretation [21].

TEE is a complex endeavor, involving motor and cognitive skills. In a study of 18 senior anesthesiologists without any ultrasound knowledge, Matyal et al. examined the impact of web-based ultrasound didactics and biweekly 90-min hands-on sessions with a TEE simulator for 4 weeks (13 target cut planes were taught). Weekly evaluation of practical skills with kinematic analysis of probe motion [22] depicted a progressive decrease in peak movements and path lengths over the 4 weeks of training, and the results at the final evaluation were close to those obtained by the experts. This three-dimensional anatomic approach explains the higher score observed in our interventional group, especially supported by an increase in practical skills. The possibility of obtaining a double image simultaneously showing the ultrasound image and the anatomic representation with the ultrasound beam also enables trainees to assimilate more accurately probe movement skills and probe positioning regarding the surrounding anatomic structures [23, 24].

A few studies of the bedside transferability of acquired skills have suggested that a TEE simulator could improve the training process [19, 20, 25–27], but were unclear about the real ability of physicians to obtain and correctly interpret images over a large range of pathological cases in real patients. They were mainly done as pretest/posttest procedures, probably due to the lack of any scoring system for evaluation of trainees at the bedside. Even a randomized study in 46 anesthesia residents (80-min TTE training using a simulator after videos and tutorial) found no significant improvement in performance in image acquisition and posttest evaluation when using a simulator [20]. Only one randomized study performed in 42 anesthesia residents reported significantly higher image quality in ten preselected standard views in real patients, especially for younger residents, after using the simulator [28]. Thanks to our previously validated scoring system, which was reported as discriminatory and sensitive to change [12], our study shows that a TEE simulator could be used to train intensivists in the use of TEE in the ICU since all evaluations were performed on real patients with hemodynamic instability. Interestingly, although we mainly reported a significant improvement in the interventional group for acquisition of practical skills, i.e., the ability of trainees to record the main views, we also found a significant improvement in technical skills, i.e., the ability of trainees to measure simple hemodynamic parameters accurately, which makes sense since technical skills are very frequently related to practical skills. These two aspects represent little more than half of the scoring system (22/40) and, interestingly, we found no significant difference between the interventional and control groups in the evaluation or interpretation skills, which are related to the ability of trainees to interpret the examination adequately and propose the right treatment. We may assume that these two components are less reliable with the use of a simulator, especially with our approach in which we used the simulator to help in the acquisition of normal images in the absence of pathology.

How inclusion of simulation will impact on a standardized curriculum has yet to be evaluated formally. The huge between-group difference we observed in the number of supervised TEE examinations required for acquisition of competency was surprising. It can in part be explained by the fact that the practical and technical skills parts represent more than half of our overall scoring system, as discussed above. However, we cannot at this point recommend the use of only 14 supervised TEE examinations for acquisition of ICU TEE competency when using a simulator, when the current recommendation is 35 [13]. Our results only support the recommendation, made by international experts in the very last document of the European Society of Intensive Care Medicine, to include TEE simulation in the training process for advanced CCE [8]. Damp et al. [11] compared fellow cardiologists, some of whom completed standard TEE training and others who were also trained with TEE simulator, while they performed bedside TEE on real patients. Like us, they found that cardiologists in the simulator group acquired a very significantly higher number of views without any assistance with much lower variability among trainees [11]. Damp et al. [11] also observed a trend toward a shorter overall TEE duration for cardiologists trained with the simulator, whereas there was no correlation between the time spent on the simulator and the evaluation scores. Previous studies on TEE simulation considered that a median 1-h simulator training is sufficient to note significant results in skills acquisition [20, 25, 29, 30]. The study by Damp et al., like ours, should help define in the future how simulation sessions have to be included in the training program of diplomas in advanced CCE.

Our study has some limitations. First, the interventional and control groups were not studied during the same period, since we compared the current period using a simulator with a previous evaluation of training not using a simulator [13]. We can’t exclude that trainees in the interventional group would have greater baseline knowledge of ultrasound than trainees in the control group included several years before. However, we used exactly the same validated scoring system to grade the ability of trainees to use and interpret ICU TEE examinations for hemodynamic evaluation. Moreover, the same expert centers also participated in the study with the same supervisors in the two periods. Second, as a consequence of the first limitation, we did not randomize trainees in the two groups. However, as shown in the results, trainees did not differ between the two periods, but we cannot exclude recruiting bias of trainees and also of the patients evaluated to grade the trainees. Third, although the simulator improved training, it acted mainly as an “accelerator” since at the end of the 6 months of training we did not note any difference between the two groups. Fourth, the supervisors have participated in training the residents and there could be a potential bias in favor of a higher score in the interventional group. Finally, our scoring system was especially developed and validated for full hemodynamic evaluation at the bedside in critically ill patients and our results cannot be extrapolated to other more specific cardiological situations.

## Conclusion

To the best of our knowledge, this is the first time that the inclusion of a TEE virtual-reality simulator in standardized ICU TEE training improved the learning curve of novices. Such training also reduced the number of supervised bedside TEE examinations needed to achieve competency in hemodynamic evaluation in the ICU. How and to what extent this kind of approach can be included in standardized curricula remain to be evaluated.

## Additional file

10.1186/s13613-016-0132-x Screenshot from TTE simulator with augmented reality image on the left and the simulated B-mode TTE image on the right.
