# Supplementary figures and images for: The use of computerized echocardiographic simulation improves the learning curve for transesophageal hemodynamic assessment in critically ill patients
Source: Ann Intensive Care. 2016 Apr 7;6:27. doi: 10.1186/s13613-016-0132-x (PMC4824699; doi:10.1186/s13613-016-0132-x)

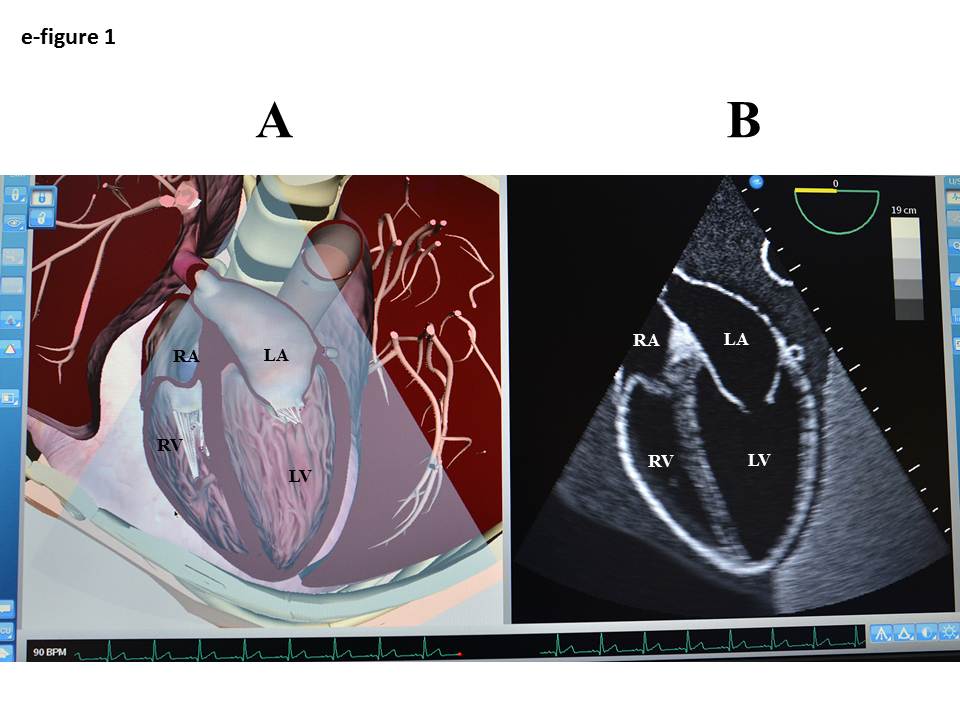

Supplement: Supplementary file 1 — 10.1186/s13613-016-0132-x Screenshot from TTE simulator with augmented reality image on the left and the simulated B-mode TTE image on the right. [file 13613_2016_132_MOESM1_ESM.jpg]
